# Supplementary material for: Diaphragm Fatigue in SMNΔ7 Mice and Its Molecular Determinants: An Underestimated Issue
Source: Int J Mol Sci. 2023 Oct 6;24(19):14953. doi: 10.3390/ijms241914953 (PMC10574014; doi:10.3390/ijms241914953)
Supplement: Supplementary file 1 [file ijms-24-14953-s001.zip › ijms-2632853-supplementary.pdf]

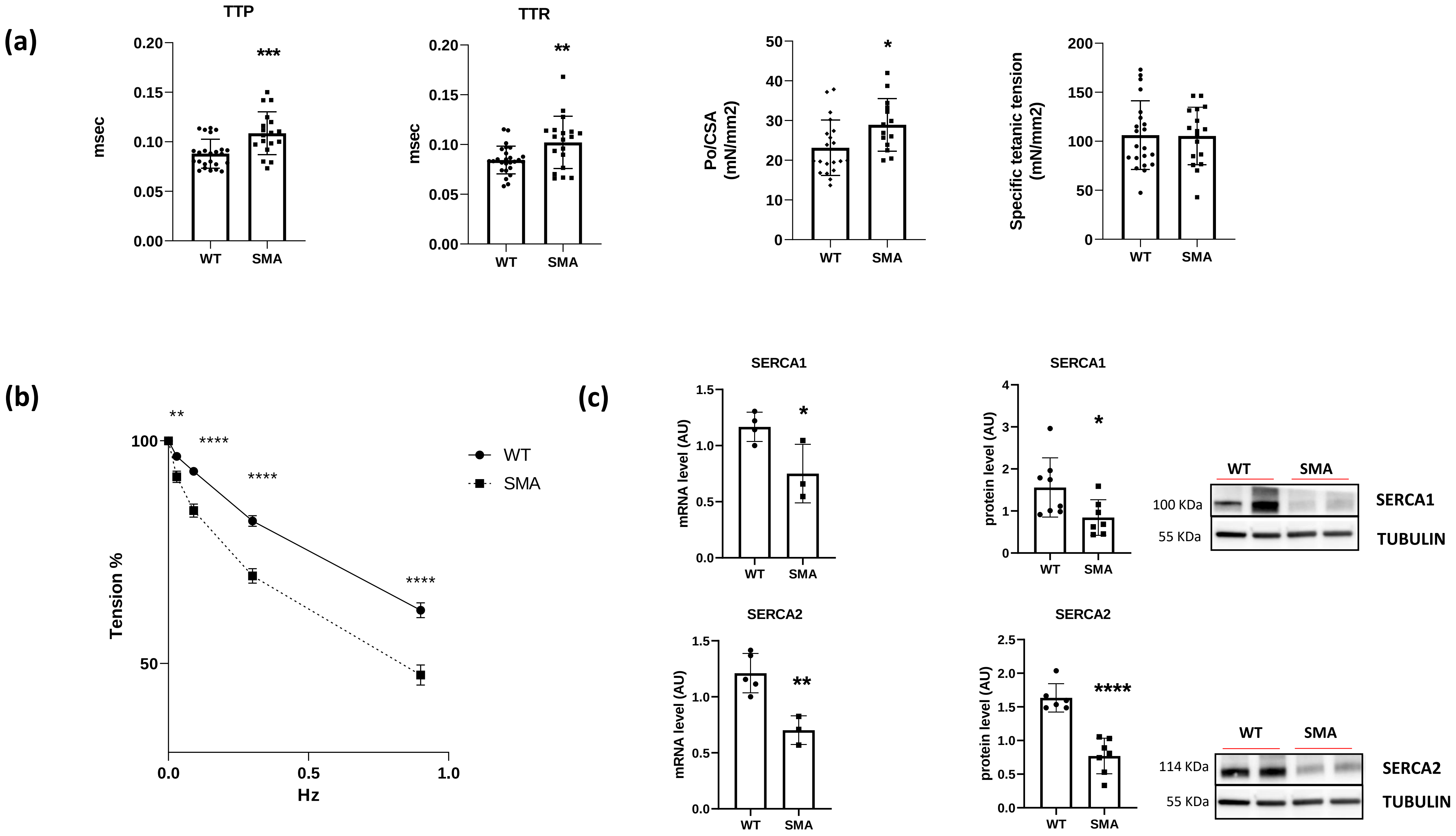

(a)
TTP
msec
0.20
0.15
0.10
0.05
0.00
***
WT
SMA
TTR
msec
0.20
0.15
0.10
0.05
0.00
**
WT
SMA
Po/CSA (mN/mm2)
50
40
30
20
10
0
*
WT
SMA
Specific tetanic tension (mN/mm2)
200
150
100
50
0
WT
SMA
(b)
Tension %
100
50
**
****
****
****
0.0
0.5
1.0
Hz
WT
SMA
(c)
SERCA1
mRNA level (AU)
1.5
1.0
0.5
0.0
*
WT
SMA
SERCA1
protein level (AU)
4
3
2
1
0
*
WT
SMA
WT
SMA
100 KDa
55 KDa
SERCA1
TUBULIN
SERCA2
mRNA level (AU)
1.5
1.0
0.5
0.0
**
WT
SMA
SERCA2
protein level (AU)
2.5
2.0
1.5
1.0
0.5
0.0
****
WT
SMA
WT
SMA
114 KDa
55 KDa
SERCA2
TUBULIN

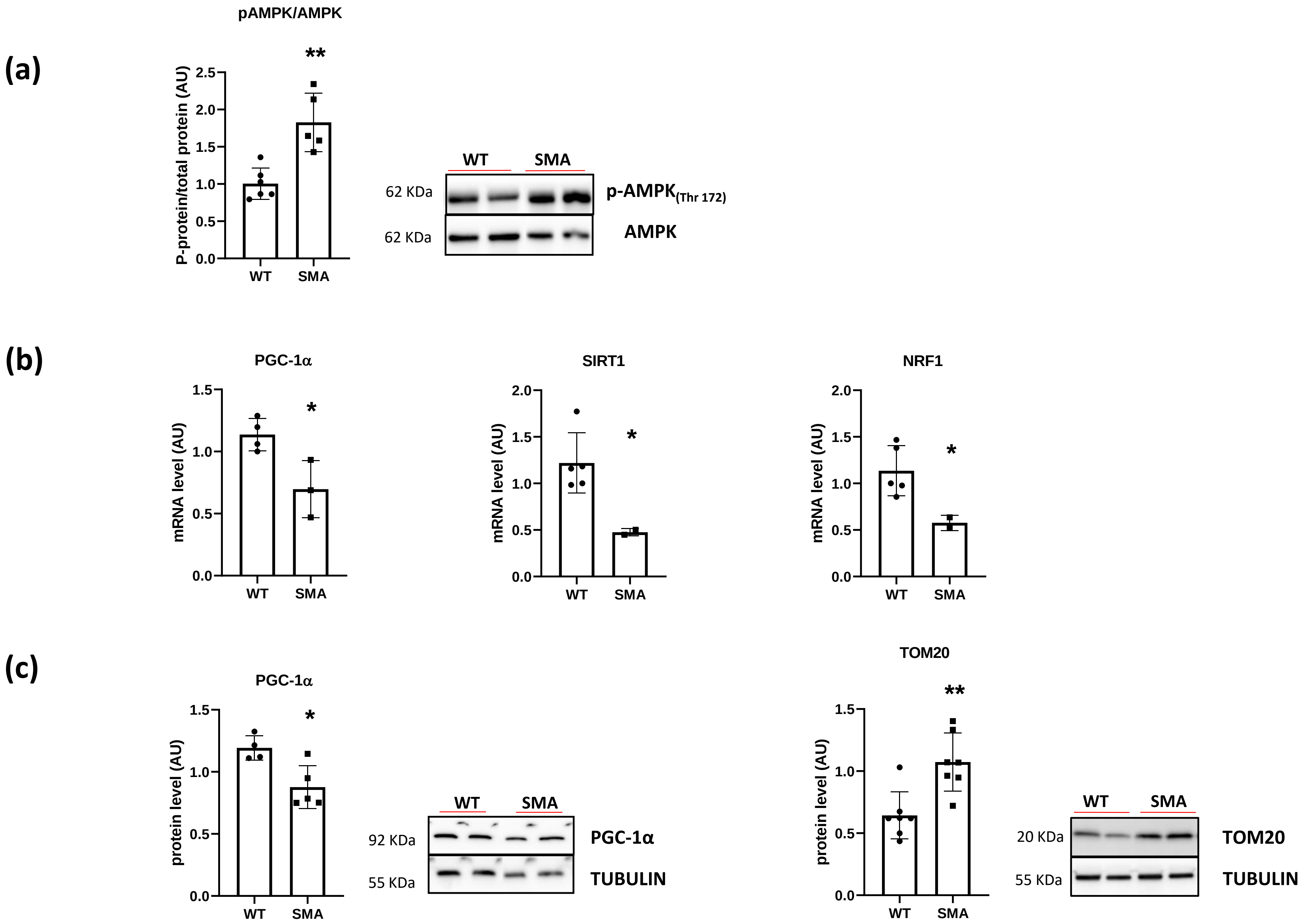
(a)
pAMPK/AMPK
**
P-protein/total protein (AU)
2.5
2.0
1.5
1.0
0.5
0.0
WT
SMA
WT
SMA
62 KDa
62 KDa
p-AMPK(Thr 172)
AMPK
(b)
PGC-1α
SIRT1
NRF1
mRNA level (AU)
1.5
1.0
0.5
0.0
2.0
*
WT
SMA
(c)
PGC-1α
TOM20
protein level (AU)
1.5
1.0
0.5
0.0
*
**
WT
SMA
92 KDa
55 KDa
PGC-1α
TUBULIN
20 KDa
55 KDa
TOM20
TUBULIN

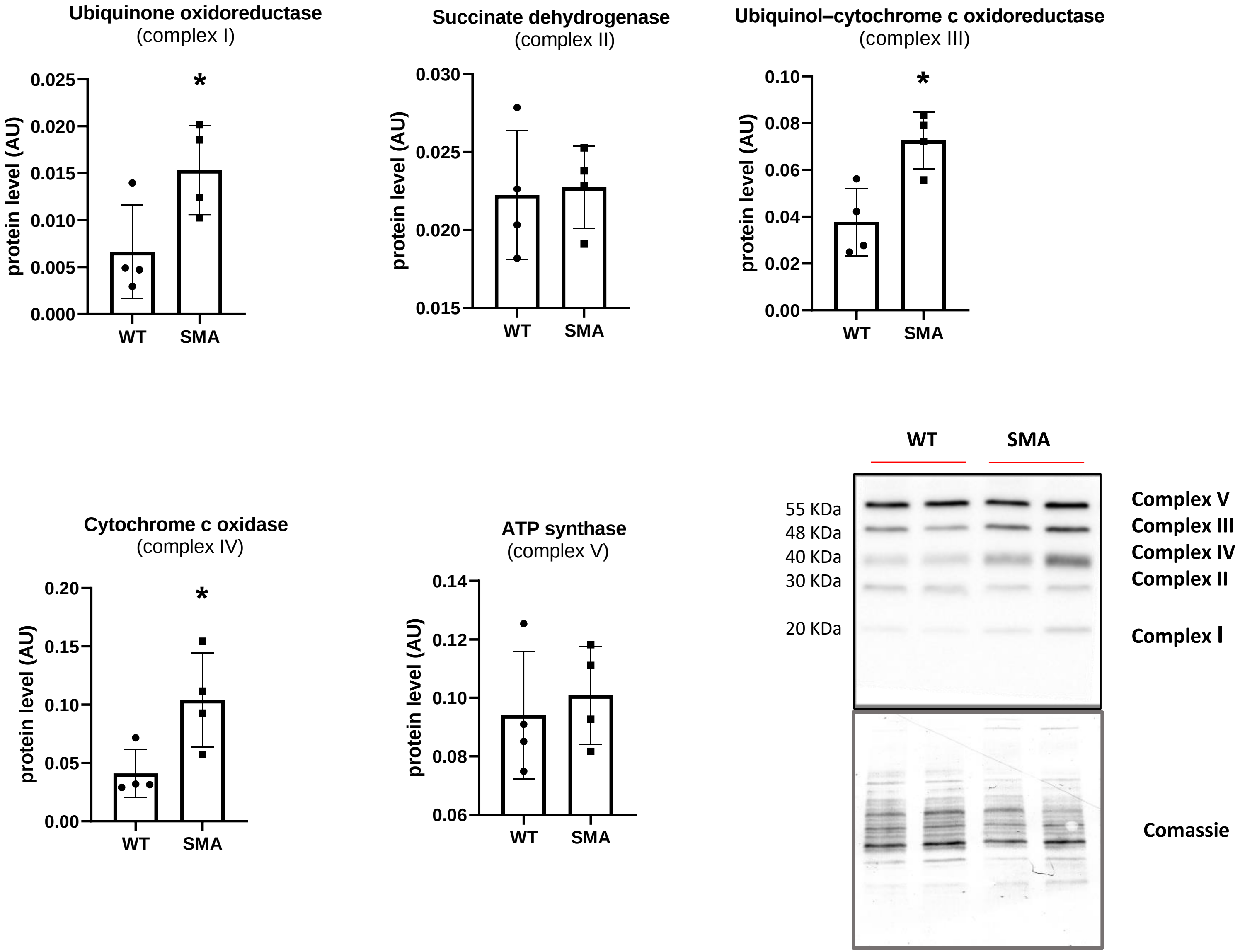
Ubiquinone oxidoreductase
(complex I)
protein level (AU)
0.025
0.020
0.015
0.010
0.005
0.000
*
WT
SMA
Succinate dehydrogenase
(complex II)
protein level (AU)
0.030
0.025
0.020
0.015
WT
SMA
Ubiquinol–cytochrome c oxidoreductase
(complex III)
protein level (AU)
0.10
0.08
0.06
0.04
0.02
0.00
*
WT
SMA
Cytochrome c oxidase
(complex IV)
protein level (AU)
0.20
0.15
0.10
0.05
0.00
*
WT
SMA
ATP synthase
(complex V)
protein level (AU)
0.14
0.12
0.10
0.08
0.06
WT
SMA
WT
SMA
55 KDa
48 KDa
40 KDa
30 KDa
20 KDa
Complex V
Complex III
Complex IV
Complex II
Complex I
Comassie

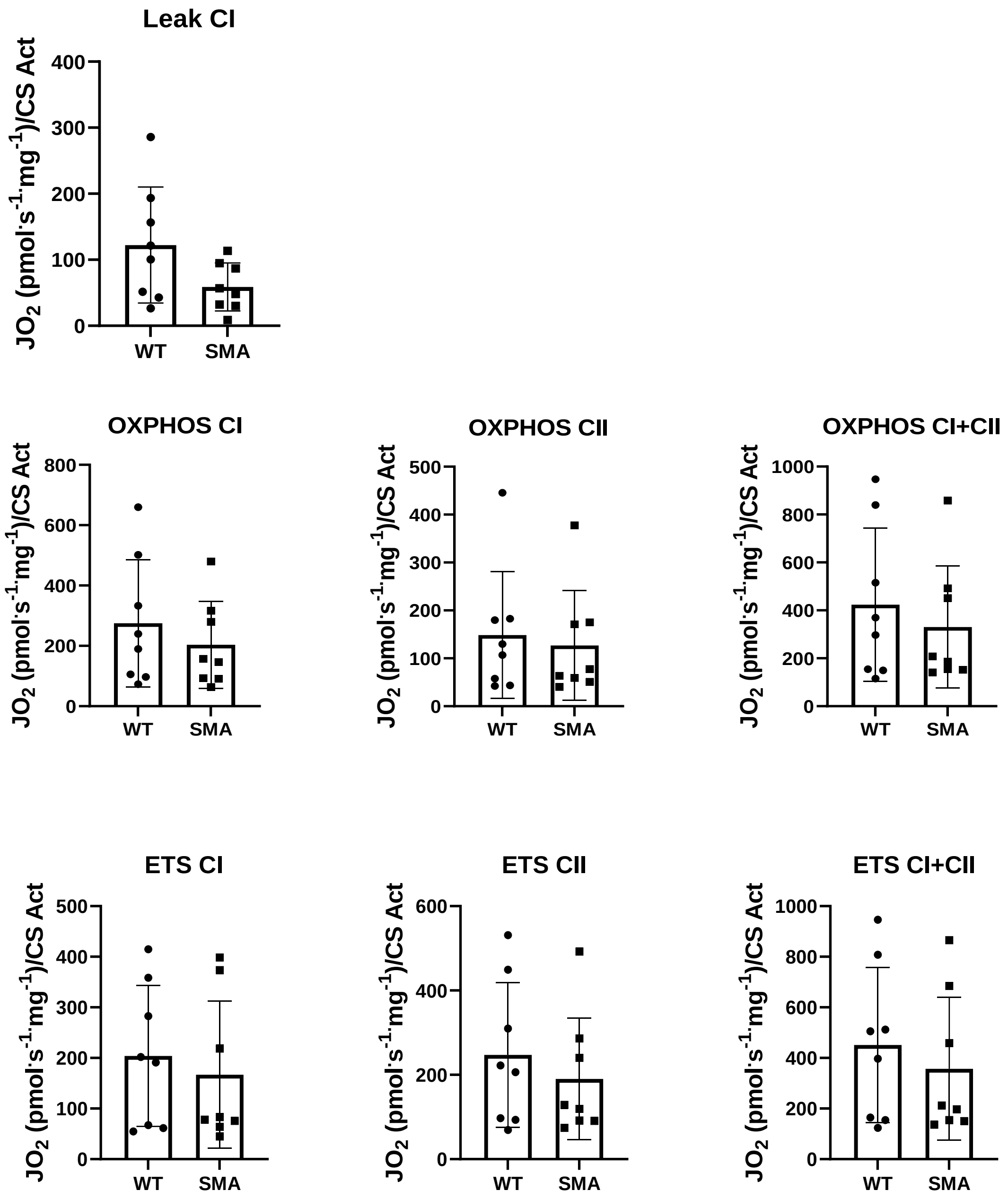
Leak CI
JO2 (pmol·s-1·mg-1)/CS Act
400
300
200
100
0
WT
SMA
OXPHOS CI
800
600
400
200
0
OXPHOS CII
500
400
300
200
100
0
OXPHOS CI+CII
1000
800
600
400
200
0
ETS CI
500
400
300
200
100
0
ETS CII
600
400
200
0
ETS CI+CII
1000
800
600
400
200
0

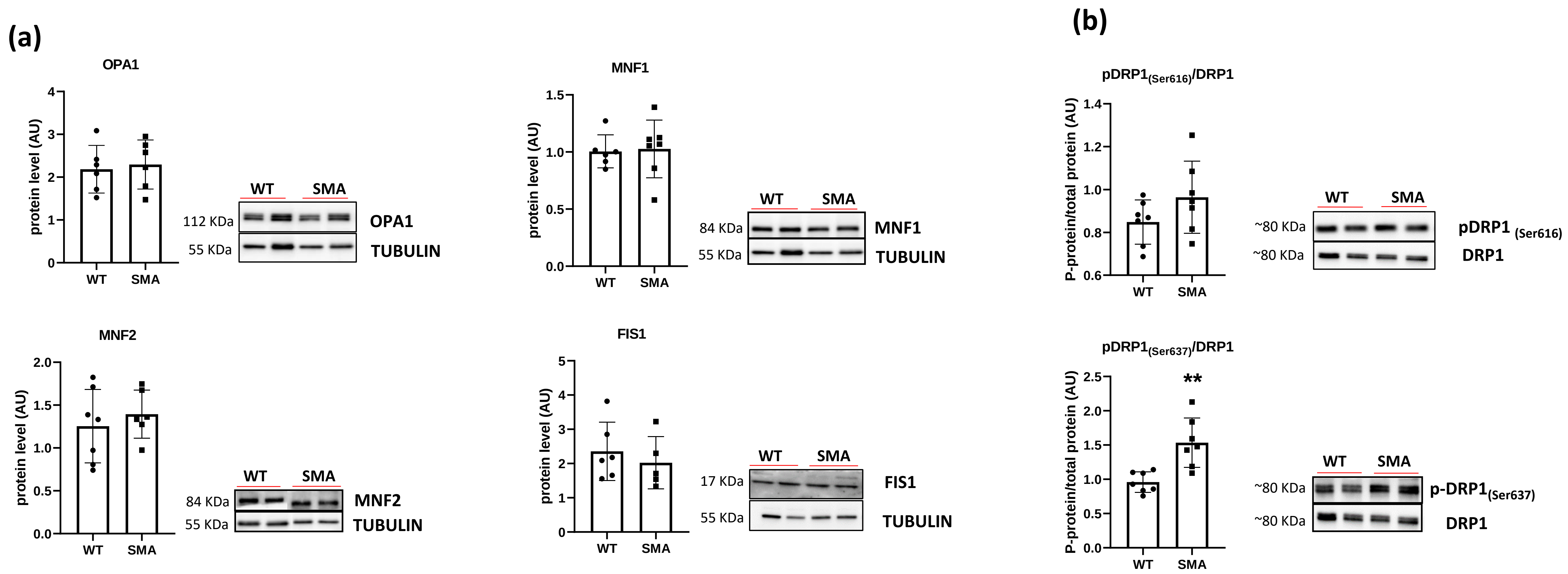
(a)
OPA1
protein level (AU)
WT
SMA
112 KDa
55 KDa
OPA1
TUBULIN
MNF1
84 KDa
55 KDa
MNF1
TUBULIN
MNF2
84 KDa
55 KDa
MNF2
TUBULIN
FIS1
17 KDa
55 KDa
FIS1
TUBULIN
(b)
pDRP1(Ser616)/DRP1
P-protein/total protein (AU)
~80 KDa
pDRP1 (Ser616)
DRP1
pDRP1(Ser637)/DRP1
**
p-DRP1(Ser637)
DRP1

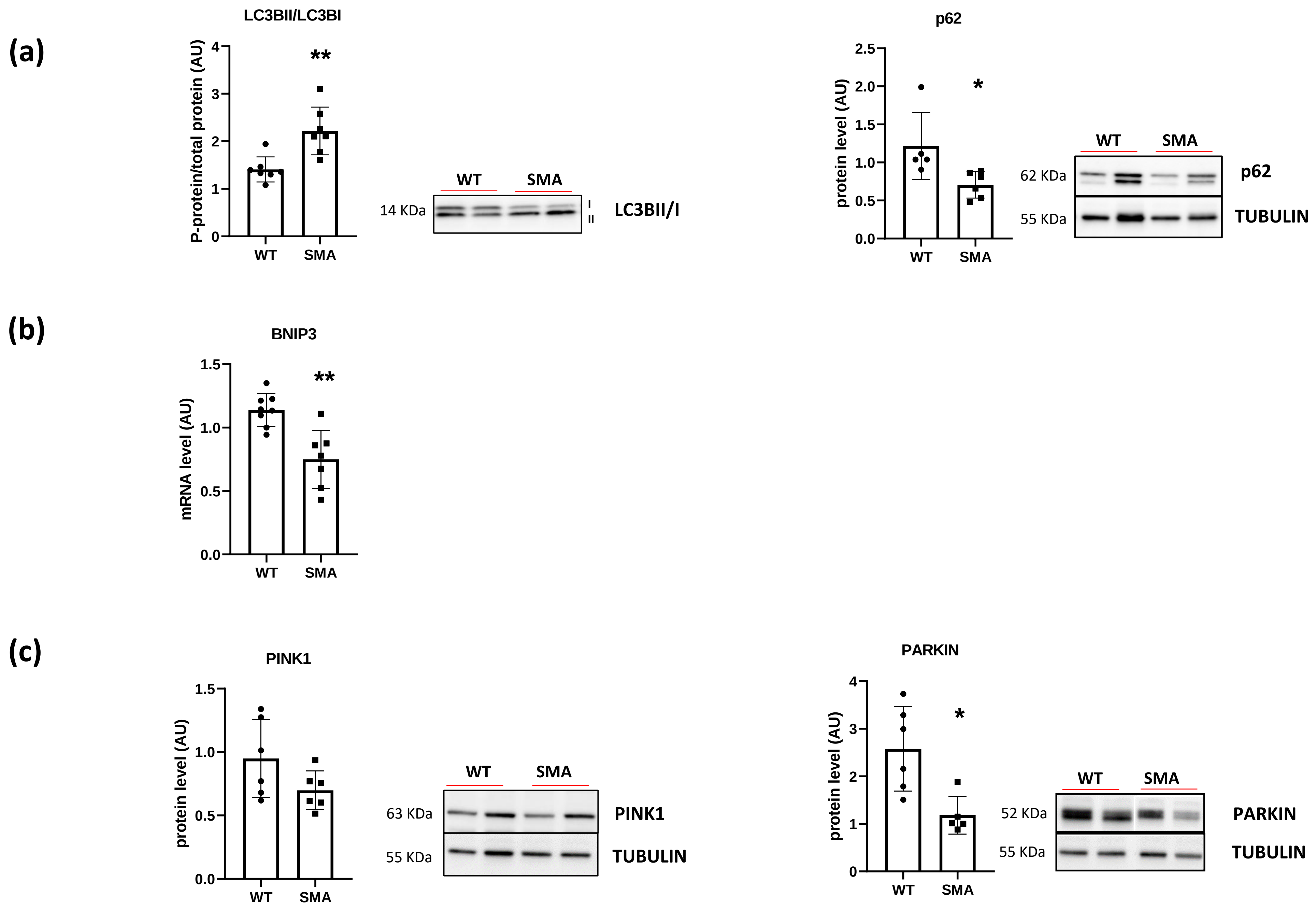
(a)
LC3BII/LC3BI
P-protein/total protein (AU)
**
WT
SMA
WT
SMA
14 KDa
I
II
LC3BII/I
p62
protein level (AU)
*
WT
SMA
WT
SMA
62 KDa
p62
55 KDa
TUBULIN
(b)
BNIP3
mRNA level (AU)
**
WT
SMA
(c)
PINK1
protein level (AU)
WT
SMA
WT
SMA
63 KDa
PINK1
55 KDa
TUBULIN
PARKIN
protein level (AU)
*
WT
SMA
WT
SMA
52 KDa
PARKIN
55 KDa
TUBULIN

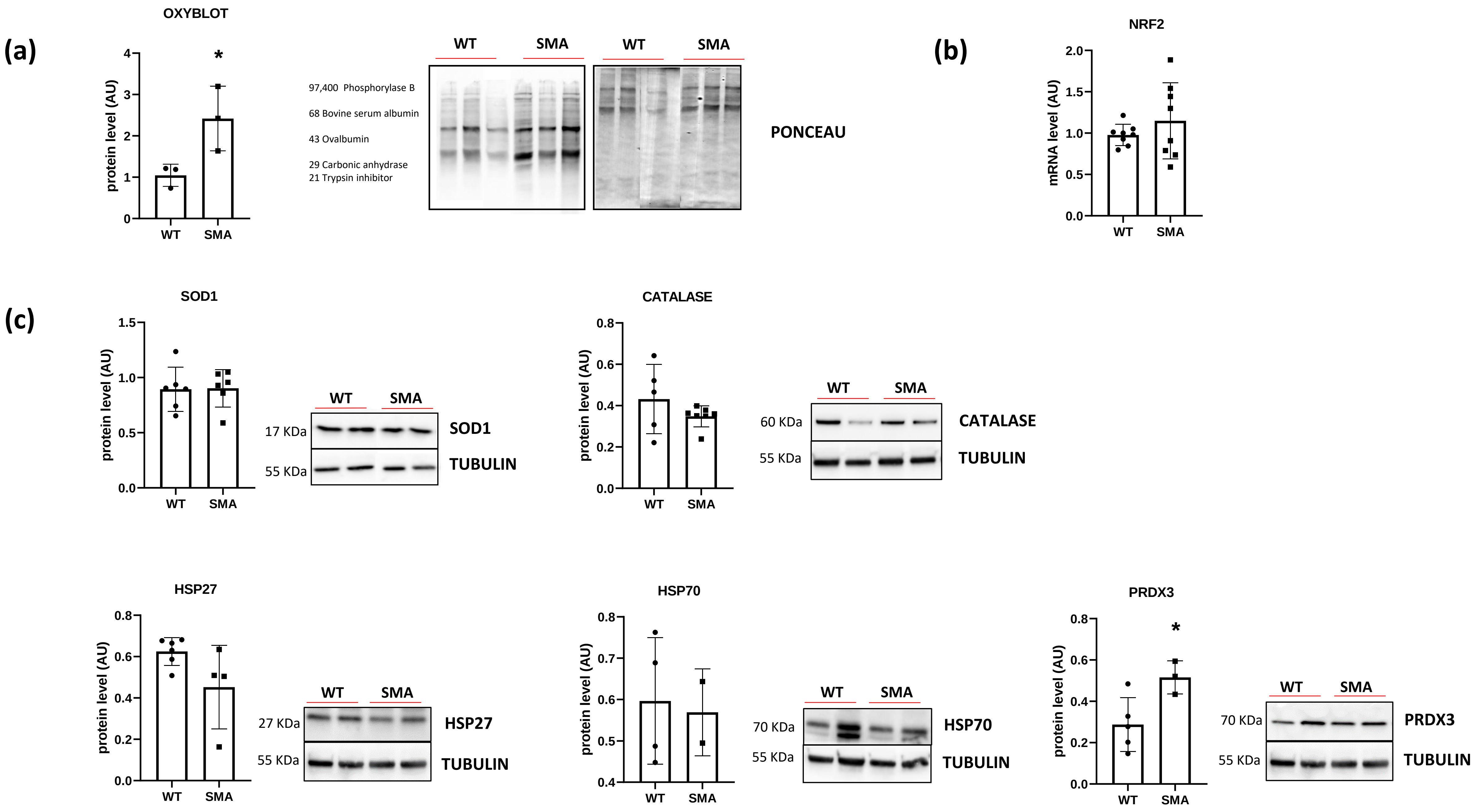
(a)
OXYBLOT
protein level (AU)
*
WT
SMA
97,400 Phosphorylase B
68 Bovine serum albumin
43 Ovalbumin
29 Carbonic anhydrase
21 Trypsin inhibitor
PONCEAU
(b)
NRF2
mRNA level (AU)
(c)
SOD1
17 KDa
55 KDa
TUBULIN
CATALASE
60 KDa
HSP27
27 KDa
HSP70
70 KDa
PRDX3

(a)

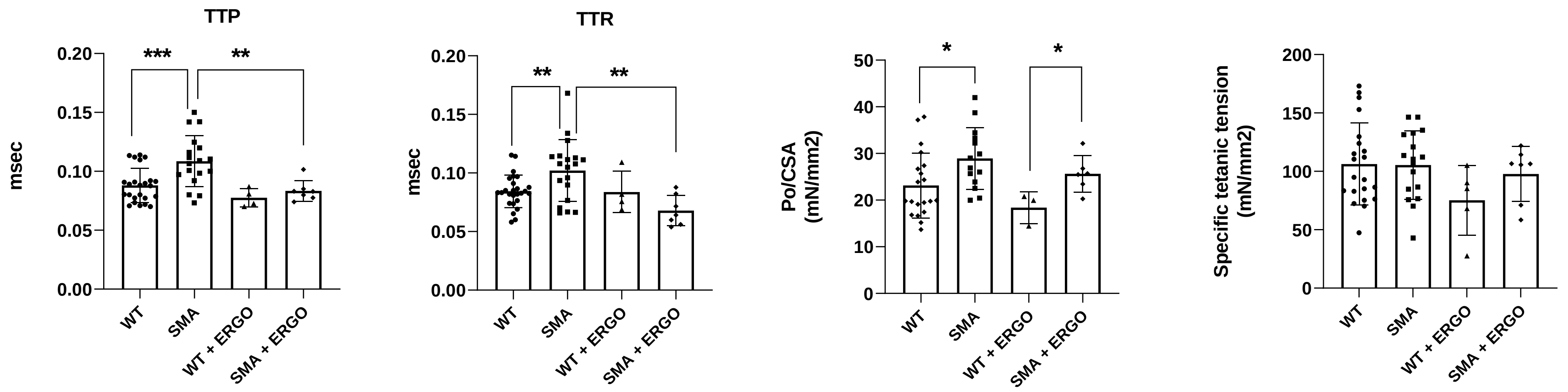
TTP
TTR
msec
0.20
0.15
0.10
0.05
0.00
***
**
*
Po/CSA (mN/mm2)
Specific tetanic tension (mN/mm2)
50
40
30
20
10
0
200
150
100
WT
SMA
WT + ERGO
SMA + ERGO

(b)

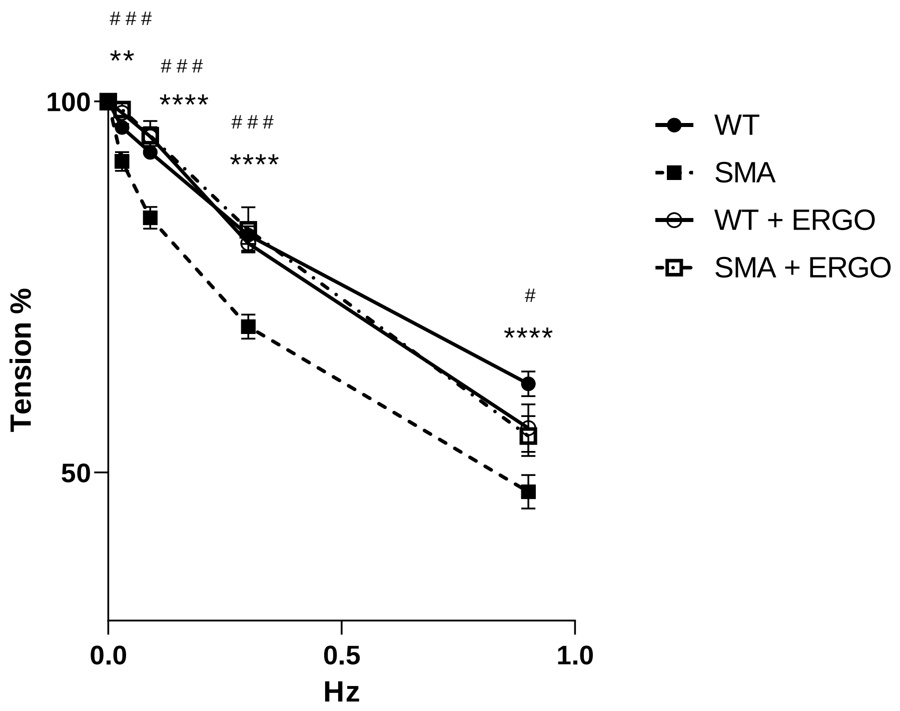
###
**
###
****
#
Tension %
100
50
0.0
0.5
1.0
Hz
WT
SMA
WT + ERGO
SMA + ERGO

Figure S1

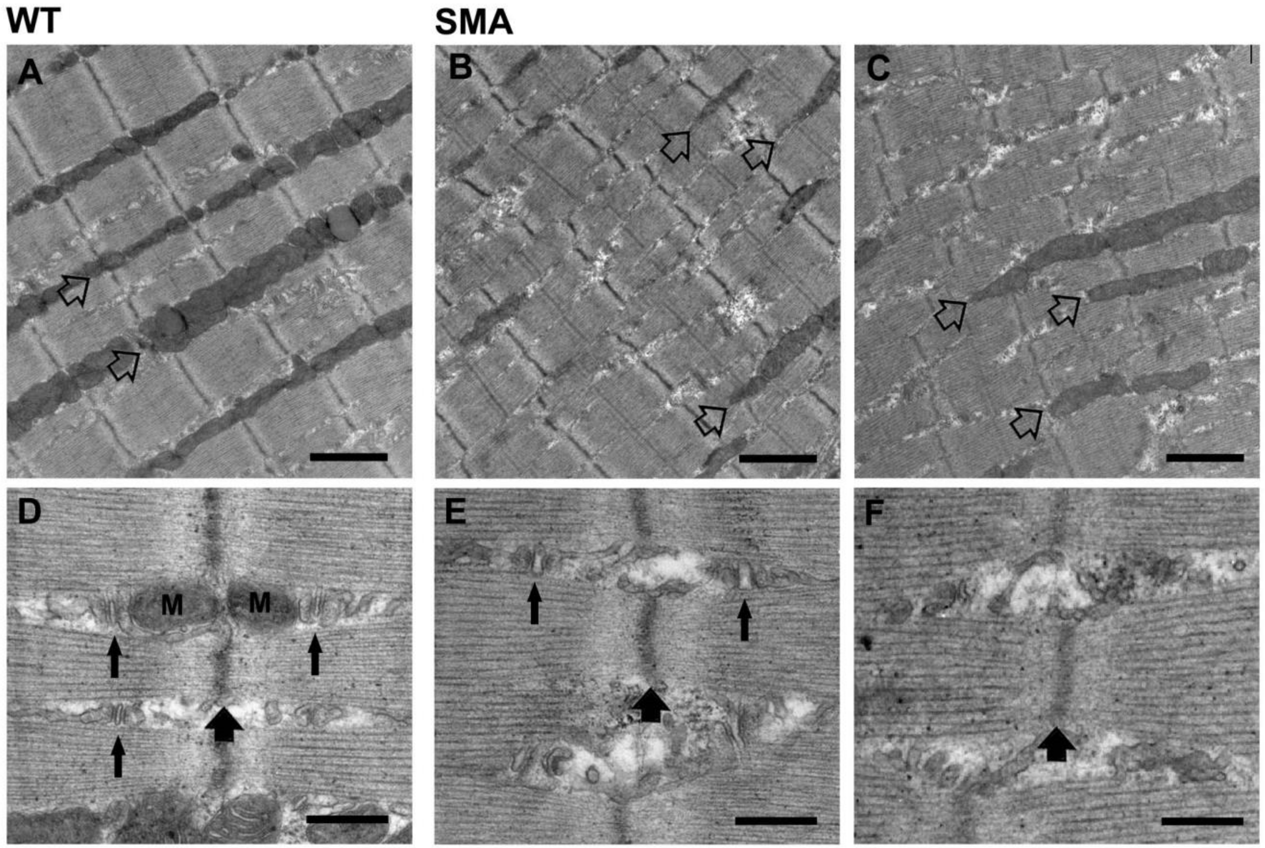

Fig. S1. Mitochondria and CRUs distribution in muscle fibers of diaphragm at 11 days of age . Representative EM images from WT (A and D) and SMA (B-F). *Labeling:* large empty arrows point to rows of mitochondria columns; small black arrows point to CRUs; large black arrows point to Z-line; M is for mitochondria. *Scale bars: A, B, and C: 2 mm; D, E and F: 0.5 mm.*

Figure S2

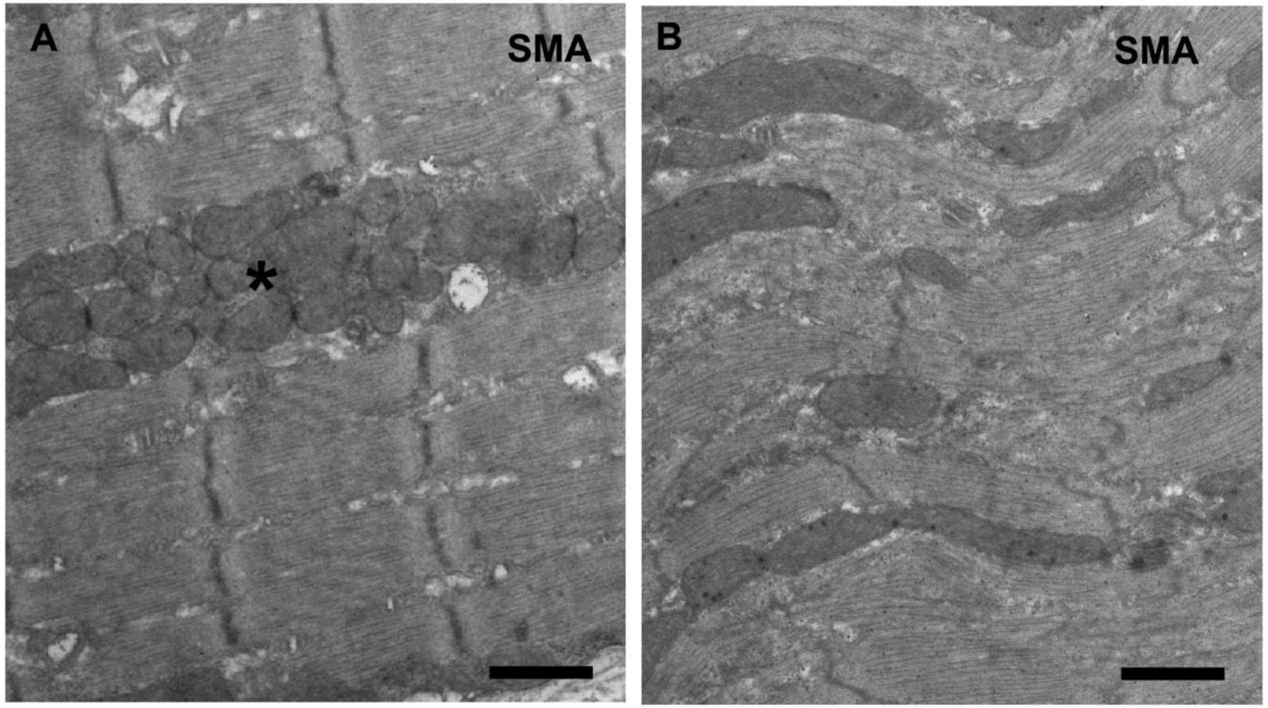

Fig. S2. Additional ultrastructural observations of diaphragm fibers at 11 days of age from SMA. Representative EM images from SMA fibers showing a large cluster of mitochondria (A, asterix) and of a completely degenerated fiber (B). *Scale bars: A and B: 1 mm.*
